# Clinical manifestation of multiple wasp stings with details of whole transcriptome analysis: Two case reports

**DOI:** 10.1097/MD.0000000000024492

**Published:** 2021-01-29

**Authors:** Wei-Fan Ou, Wei-Hsuan Huang, Hsien-Fu Chiu, Yan-Chiao Mao, Mei-Chin Wen, Cheng-Hsu Chen, Sheng-Jou Hung, Ming-Ju Wu, Chieh-Liang Wu, Wen-Cheng Chao

**Affiliations:** a Department of Internal Medicine; b Division of Nephrology, Department of Internal Medicine; c Division of Clinical Toxicology, Department of Emergency Medicine; d Department of Pathology and Laboratory Medicine, Taichung Veterans General Hospital; e Department of Life Science, Tunghai University; f Department of Medical Research, Taichung Veterans General Hospital; g School of Medicine, Chung Shan Medical University; h Rong Hsing Research Center for Translational Medicine, Institute of Biomedical Science, College of Life Science, National Chung Hsing University; i Graduate Institute of Clinical Medical Science, School of Medicine, China Medical University; j Department of Critical Care Medicine, Taichung Veterans General Hospital; k Department of Industrial Engineering and Enterprise Information; l Department of Computer Science, Tunghai University, Taichung, Taiwan.

**Keywords:** acute respiratory distress syndrome, case report, plasmapheresis, RNA-Seq, wasp

## Abstract

**Introduction:**

Multiple wasp stings is an emergency result from systemic reactions to the toxin with a wide range of manifestations, and we presented 2 patients with distinct clinical and transcriptomic findings.

**Patient concerns:**

Two patients without systemic disease presented with nearly 90 painful papules after attacked by a swarm of wasps (*Vespa basalis*).

**Diagnosis:**

Patient 1 was a 44-year-old healthy male whose clinical manifestations mainly comprised hemolysis, hepatic injury, rhabdomyolysis, and acute kidney injury. Patient 2 was a 49-year-old healthy female who presented with severe acute respiratory distress syndrome (ARDS) in addition to certain clinical manifestations that were also found in patient 1. We used ribo- nucleic acid sequencing (RNA-Seq) to characterize the inflammatory responses of 2 patients with distinct clinical manifestations after multiple wasp stings.

**Interventions:**

Both 2 patients received 5 sessions of plasmapheresis, and patient-1 further received mechanical ventilation for 8 days as well as 8 sessions of hemodialysis until day 17.

**Outcomes:**

Both patients recovered uneventfully after the aforementioned management. We used RNA-Seq to demonstrate a largely regulated neutrophil-predominated immune response in patient 1. In patient 2, we found a profound neutrophilc response on week 1 and a robust neutrophilic as well as pro-inflammatory responses on week 2. Furthermore, we found increased expression of signals that were associated with renal system process on week 2.

**Conclusion:**

In conclusion, we report 2 patients who manifested with shared and distinct presentations after an attack by the same swarm of wasps. Both patients had hemolysis, rhabdomyolysis, hepatic injury and acute kidney injury, and 1 patient had ARDS. The whole transcriptomic analyses were consistent with the distinct clinical manifestation, and these results suggest the potential of RNA-Sequencing to disentangle complex inflammatory responses in patients with multiple wasp stings. Plasmapheresis and corticosteroid were administered to both patients and case 2 also underwent 8 sessions of hemodialysis.

## Abstract 2

Supplemental Digital Content is available in the text

## 1 Introduction

Wasp stings are an increasing public health problem worldwide, particularly in Asian countries.^[1–4]^ The wasp sting mainly manifests with inflammatory responses potentially leading to anaphylaxis, and more than 20 stings may be fatal due to the various toxic reactions to the venom, so-called envenomation, which include catastrophic organ damage, hemolysis, rhabdomyolysis, acute kidney injury, hepatic injury, and rarely, acute respiratory distress syndrome (ARDS).^[4–6]^ Given the complexity of wasp venom and distinct individual responses, there is a wide range of clinical presentations in patients with envenomation resulting from multiple wasp stings. RNA-Sequencing (RNA-Seq) is currently an essential strategy to address complex diseases, including kidney and ARDS, which disrupts the pathophysiological homeostasis result from complex, numerous, intertwined, subcellular biological events.^[7–9]^ Herein, we present 2 patients attacked by a swarm of black-bellied hornets (*Vespa basalis*). They both developed hemolysis, rhabdomyolysis, and acute kidney injury (AKI), but only one of them had ARDS. We also used sequential RNA-Seq on week-1 and week-2 to characterize the distinct inflammatory features between these 2 patients.

## 2 Ethics

The present study was approved by the Institutional Review Board of Taichung Veterans General Hospital, Taiwan (IRB number: CE19395A). Written informed consent was obtained from the 2 patients for publication of this study and any accompanying images.

## 3 Case report

### 3.1 Case 1

**Figure 1:**
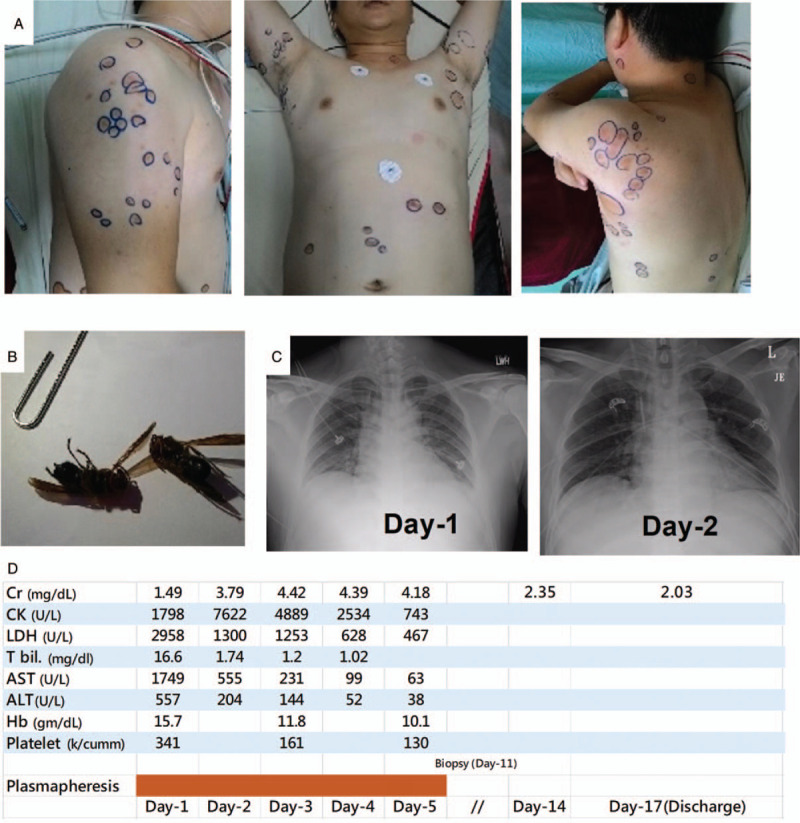
Skin lesions on the trunk (A), 2 captured dead wasps (B), serial chest X-ray (C), and serial laboratory findings and management (D) of case 1. ALT = alanine aminotransferase, AST = aspartate aminotransferase, CK = creatine kinase, Cr = creatinine, Hb = hemoglobin, LDH = lactate dehydrogenase, Plt = platelet.

**Figure 2:**
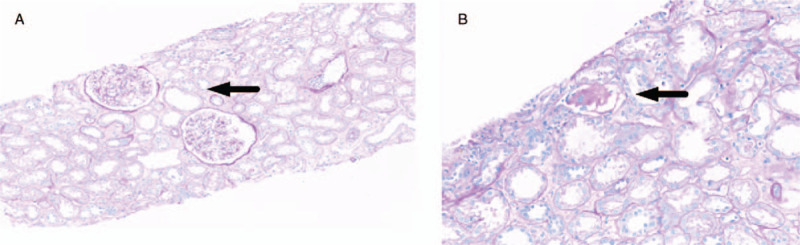
Pathological findings of the renal biopsy in case 1. Low power view showing normal glomerular, interstitial edema, and dilated tubule (arrow) (H&E staining, magnification ×50) (A); high power view showing acute tubular injury with diffuse attenuated luminal brush border and cell flattening of tubule and presence of Tamm-horsfall protein casts (arrow) in the tubule (H&E staining, magnification × 100) (B).

A 44-year-old male without systemic disease was stung by a swarm of wasps in a rural area in central Taiwan. He was brought to a local hospital within 1 hour and approximately 90 painful, red, pustular lesions were noted (Fig. 1A). After administration of anti-histamine agents and intravenous corticosteroid, he was referred to Taichung Veterans General Hospital (TCVGH) for further management nearly 8 hours after being stung. *Vespa basalis* was identified by the appearance of the 2 captured dead wasps (Fig. 1B). The patient was clear in consciousness without dyspnea, facial swelling, or hypotension. On examination, his body temperature was 36.2°C; blood pressure was 193/126 mm Hg; pulse rate was 80 beats/minute, and respiratory rate was 16/minute. The chest X-ray (CXR) was unremarkable and the electrocardiogram showed a normal sinus rhythm (Fig. 1C). The laboratory examinations revealed abnormal liver function tests, including elevated total bilirubin 16.6 mg/dl, direct bilirubin 4.39 mg/dl, aspartate aminotransferase (AST) 1749 U/L, alanine aminotransferase (ALT) 557 U/L, high lactate dehydrogenase (LDH) (2958 U/L) as well as creatine kinase (CK) 1798 U/L, and slightly elevated serum creatinine (1.49 mg/dl) (Fig. 1D). The urinalysis showed 2+ dipstick proteinuria and 5 red blood cells per high power field. Therefore, a diagnosis of multiple *Vespa basalis* stings complicated with hemolysis, rhabdomyolysis, hepatic injury, and acute kidney injury was made, and prompt plasmapheresis, 1.3 times the plasma volume, for a total of 5 sessions was applied in addition to hydration and alkalization of urine. The patient recovered well and the creatinine level reached the peak on day 4. Additionally, corticosteroid was administered due to the possible interstitial nephritis. Renal biopsy, performed on day-11, showed mainly residual acute tubular injury with Tamm-Horsfall protein casts and pigmented casts; therefore, corticosteroid was discontinued given that no evidence for further interstitial nephritis (Fig. 2). The serum creatinine was 2.03 mg/dl on discharge (day 17) and had returned to normal (1.13 mg/dl) at follow-up in the outpatient department (day 53). RNA-Seq and pathway annotation showed an increased response to bacterium on week-1 and modestly highly expressed pro-inflammatory responses, including leucocyte activation, neutrophilc degranulation, and humoral immune response (see supplemental data for detailed methodology, http://links.lww.com/MD/F603) (Fig. 4A and B).^[10]^ The result of whole transcriptome analyses appears to be consistent with the clinical manifestation with wasp toxin-associated pro-inflammatory responses, which recovered few days later after the aforementioned management.

### 3.2 Case 2

**Figure 3:**
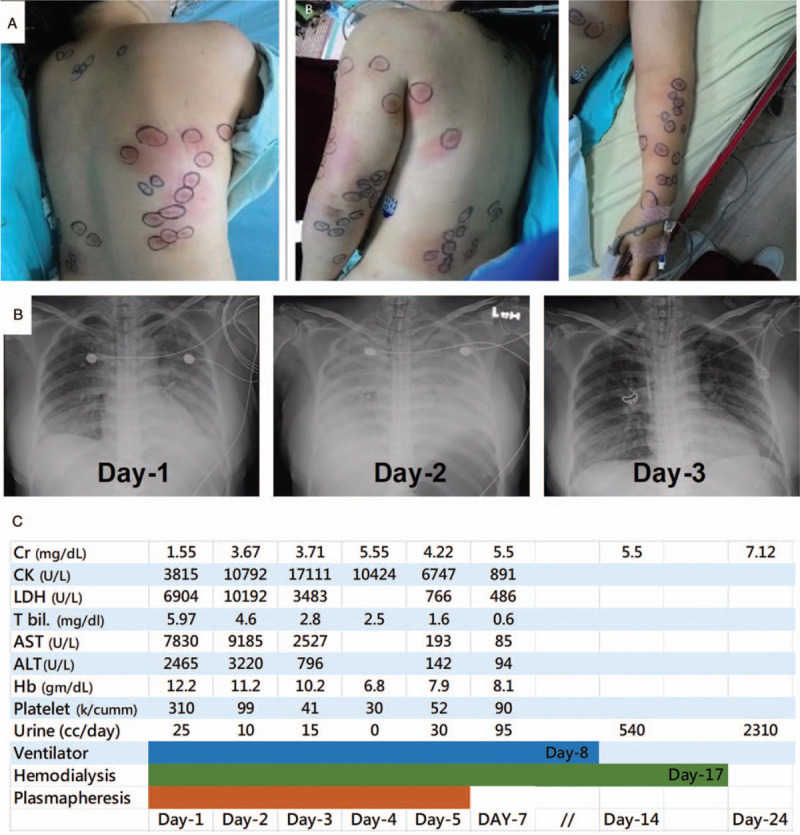
Skin lesions on the trunk (A), serial chest X-ray (B), and serial laboratory findings and management (C) of case 2. ALT =  alanine aminotransferase, AST = aspartate aminotransferase, CK = creatine kinase, Cr = creatinine, Hb = hemoglobin, LDH = lactate dehydrogenase.

**Figure 4:**
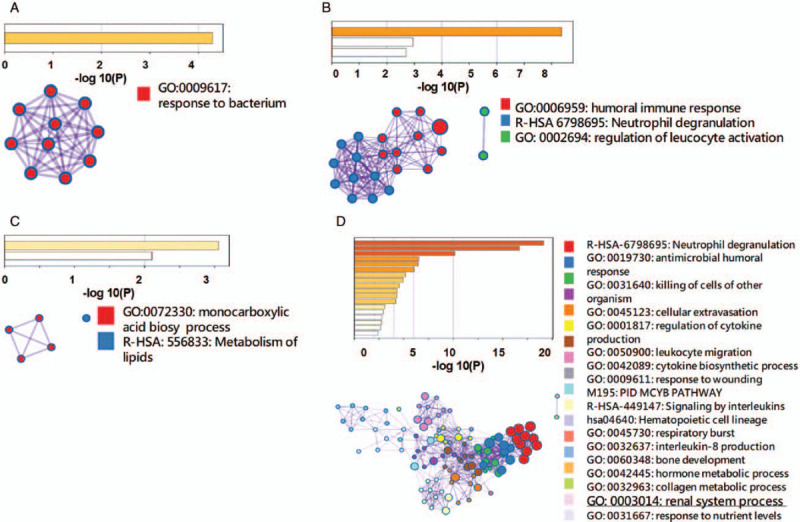
Functional enrichment of gene ontology analyses of case 1 (A: week-1; B: week-2) and case 2 (C: week-1; D: week-2) using Metascape. (Bar graph of the enriched terms of differentially expressed genes coloured according to *P* values).

A 40-year-old woman without systemic disease was stung at the same time by the same swarm of wasps as described in case 1. She was also brought to a local hospital within 1 hour and nearly 100 painful, reddish and itchy papules were noted (Fig. 3A). After administration of anti-histamine agents and intravenous corticosteroid, she was also referred to TCVGH nearly 8 hours after being stung. However, she felt breathless during the transfer. On arrival at TCVGH, she was clear in consciousness but had apparent respiratory distress. On examination, her body temperature was 37.2°C; blood pressure was 133/85 mm Hg; pulse rate was 86 beats/minute, but respiratory rate was 26/minute and SaO2 was 88% under nasal cannula 6 L/minute. Electrocardiogram revealed normal sinus rhythm, and the initial CXR showed mildly increased infiltration in bilateral lung fields (Fig. 3B). The initial laboratory examinations also showed abnormal liver function tests (total bilirubin 5.97 mg/dl, AST 7830 U/L, ALT 2465 U/L), high LDH 6904 U/L, as well as CK 3815 U/L, and increased level of serum creatinine (1.55 mg/dl) (Fig. 3C). Urinalysis showed 3+ dipstick proteinuria and 5 to 10 red blood cells per high power field. The patient had progressive shortness of breath and desaturation despite the application of intravenous corticosteroid and non-rebreathing oxygen mask and was intubated approximately 3 hours after admission to TCVGH. The follow-up CXR showed rapidly developed bilateral alveolar lesions and the arterial blood gas exam revealed pH of 7.26, HCO_3_ of 14.8 mEq/L, PCO2 of 33 mm Hg, and PaO2 of 57 mm Hg under FiO_2_ 100% (ratio of PaO_2_/FiO_2_: 57). Therefore, severe acute respiratory distress syndrome (ARDS) was diagnosed and protective ventilation with low tidal volume (7 ml/kg predicted body weight) and high positive end-expiratory pressure (PEEP) (14 cmH_2_O), sedative agent, and neuromuscular blockade were applied. Anuria was found after admission and hemodialysis was promptly applied on day 1. The patient tolerated the HD well and had stable blood pressure. FiO_2_ was then steadily reduced to 50% on day 2. As in case 1, plasmapheresis was performed, with 1.3 times the plasma volume in each session, for a total of 5 sessions (Fig. 3C). Her respiratory condition improved gradually and was extubated uneventfully on day 8. The levels of CK, AST, ALT, and LDH reached the peak on day 2 and recovered after the aforementioned management, including plasmapheresis, HD, and administration of corticosteroid. Anuria persisted for nearly 10 days and the patient received HD for a total of 8 sessions till day 17. Case 2 was discharged on day 24 with creatinine 7.12 mg/dl, and the creatinine returned to normal (Cr:1.16) during follow-up in the outpatient department on day 71. In contrast with the largely regulated neutrophilic response in case 1, there were profound and persistent neutrophilc response in case 2. Furthermore, a number of pro-inflammatory pathways, including cytokine production, signaling by interleukins, interleukin-8 production and respiratory burst, were also highly expressed on week 2. Moreover, we identified modestly increased expressed pathways of renal system process, indicating the gradual recovery of the renal function on week 2 (Fig. 4C and D).

## 4 Discussion

Multiple wasp stings have a wide range of clinical presentations. Herein, we report 2 patients who manifested with shared and distinct presentations after an attack by the same swarm of wasps. The patients both had hemolysis, rhabdomyolysis, as well as AKI, and recovered after plasmapheresis, whereas 1 patient also had ARDS. The whole transcriptomic analyses were consistent with the clinical manifestations of the 2 patients and might be used to clarify complex inflammatory responses in patients with multiple wasp stings.

In the present study, case 2 had severe ARDS with quite a low ratio of PaO_2_/FiO_2_ (57), and the severe ARDS was managed uneventfully by protective mechanical ventilation and dry-lung strategy through prompt hemodialysis. Intriguingly, ARDS is somehow rarely reported in wasp stings. Xie et al analyzed a total of 1091 subjects hospitalized for wasp stings and found that merely 1.6% (17/1091) of patients had ARDS.^[2]^ In 1 large-scale global surveillance study, approximately one-third of patients with ARDS were somehow unrecognized.^[11]^ Hence, we think that ARDS might be under-recognized in patients with multiple wasp stings and is probably mainly recognized in patients with respiratory failure and poor oxygenation function, as we found in case 2. Additionally, we used the RNA-Seq to demonstrate a highly increased neutrophil relevant pathways, on both week-1 and week-2 in case-2. Indeed, the neutrophil influx, release of cytokine/chemokine cascades, oxidative stress, and profound inflammatory cascade have been found to be substantial in the increased epithelial and endothelial permeability as well as hypoxemia among ARDS.^[12–14]^ The consistency between clinical manifestation and transcriptomic analyses as we have shown in the present study indicates that RNA-Seq should provide comprehensive biological information to identify under-recognized ARDS although timely RNA-Seq remains a niche.

Acute kidney injury (AKI) is one of the most severe consequences of wasp stings. Wasp sting-induced AKI can be further classified into 2 types: acute tubular necrosis (ATN), resulting from rhabdomyolysis/hemolysis, and acute interstitial nephritis (AIN), which may respond to the use of corticosteroid.^[15,16]^ Both patients in this study had AKI, and case 2 required hemodialysis for 17 days (8 sessions). Case 1 received renal biopsy on day 11, and the pathological finding was consistent with residual ATN without evidence of AIN (Fig. 2). It is estimated that 21% to 58.5% of patients with wasp stings requiring hospitalization had various degrees of AKI, which was correlated with the severity of rhabdomyolysis/hemolysis.^[2,17]^ Wasp venom consists of complex biogenic components, including phospholipase-1, phospholipase-2, and hyaluronidase, and these toxins disrupt the cell membrane of red blood and skeletal cells and lead to hemolysis as well as rhabdomyolysis.^[6]^ The number of wasp stings appears to be correlated with the severity of AKI, and therefore the treating physician should be particularly vigilant in the care of patients with significant envenomation, that is, more than 20 wasp stings.^[5]^ Dhanapriya et al investigated 11 patients with wasp sting-induced AKI and found that 10 patients required hemodialysis and the mean number of hemodialysis sessions was 8.7 ± 2.8.^[15]^ Furthermore, Zhang et al analyzed 81 patients with AKI after multiple wasp stings and found that the average duration of renal replacement therapy (RRT) was 18.2 ± 8.4 days.^[18]^ The aforementioned evidence is consistent with the finding in the present study, that is, case 2 required 8 sessions of HD till day 17 after sustaining multiple wasp stings. Intriguingly, we found an increased expression of *SULF2, JCHAIN,* and *PARKAR2B* on week 2 in case 2. In detail, SULF2 protein plays a key role in glomerular homeostasis through regulating growth factor signaling.^[19]^*JCHAIN* encodes J chain of IgA and is implicated with the pathogenesis of IgA nephropathy.^[20,21]^*PRKAR2B* encodes cAMP-dependent protein kinase type II-beta regulatory subunit, and cAMP is an essential signaling molecule of aquaporin protein 1 in the kidney.^[22]^ The aforementioned renal system relevant signaling could suggest the underlying recovery of renal function, although more studies are warranted to elucidate transcriptional trajectories in patients with kidney injury result from multiple wasp stings.^[23]^

A number of modalities of RRT including intermittent hemodialysis (IHD), sustained low-efficiency hemodialysis (SLED), and continuous renal replacement therapy (CRRT), mainly in the form of continuous veno-venous hemofiltration (CVVH), have been applied in patients with wasp stings requiring RRT.^[18,24]^ Compared with IHD, CVVH was found to be more effective for reducing plasma levels of myoglobin, CK, LDH, bilirubin, as well as creatinine, and incidence of oliguria and the duration of hospitalization were hence reduced.^[25]^ Ye et al analyzed 35 patients with multiple organ dysfunction after wasp stings and reported that SLED, CVVH, and IHD had similar renal recovery and survival rates, and that patients who underwent HD were more likely to have hypotension and arrhythmia.^[6]^ Therefore, SLED/CVVH might be considered, particularly for patients who are unstable, such as those with hypotension or arrhythmia. It should be emphasized that the removal of toxic components in patients with wasp envenomation is of paramount importance due to the lack of anti-venom for wasp stings. Therefore, PE has been introduced in the management of wasp sting-induced AKI as plasmapheresis may reduce circulating venom and remove the circulating inflammatory mediators in patients with envenomation.^[26]^ Zhang et al analyzed 81 patients with multiple wasp stings who received RRT and found a similar mortality rate among patients receiving CVVH, CVVH with plasmapheresis, or IHD-alone; however, the kidneys of patients in the IHD-alone group appeared to have a longer recovery time compared with those of patients in the CVVH and CVVH with plasmapheresis groups (41.6 ± 8.1 days, 31.9 ± 8.5 days, 28.6 ± 9.4 days, respectively).^[6,18]^ The aforementioned evidence reflect the heterogeneity of multiple wasp stings complicated with AKI, and plasmapheresis with CVVH appeared to be a reasonable combinational modality, particularly in patients with unstable conditions.^[6]^ In the present study, both of our patients received 5 sessions of plasmapheresis. Case 2 tolerated IHD well and achieved a stable condition, so CVVH was not applied.

In conclusion, 2 patients with multiple swap stings inflicted by the same swarm of wasps shared a number of clinical manifestations, but there were also notable differences between them. Both patients had hemolysis, rhabdomyolysis, hepatic injury, and AKI, and one of the patients additionally developed ARDS. The transcriptomic analyses were consistent with the clinical manifestation of the 2 patients with multiple wasp stings and may potentially be used to disentangle complex inflammatory responses in patients with multiple wasp stings. The patients’ renal function recovered well following application of plasmapheresis and administration of corticosteroid. Case 1 recovered without HD, whereas case 2 required 8 sessions of HD till day 17. Further studies are warranted to elucidate the cellular mechanism in patients with multiple wasp stings.

## Acknowledgments

We thank the multidisciplinary medical staff at TCVGH for their contributions in the diagnosis and management of the 2 patients presented in this case report.

## Author contributions

Conceived and designed the study: HFC, CHC, MJW, CLW, and WCC. Acquired the data: WFO, WHH, SJH, MCW, and WCC. Contributed to the materials: WFO, HFC, YCM, and WCC. Wrote the manuscript: WFO, MJW, and WCC. All authors have read and approved the manuscript.

**Conceptualization:** Wei-Fan Ou, Hsien-Fu Chiu, Cheng-Hsu Chen, Ming-Ju Wu, Chieh-Liang Wu, Wen-Cheng Chao.

**Data curation:** Wei-Fan Ou, Wei-Hsuan Huang, Mei-Chin Wen, Sheng-Jou Hung, Wen-Cheng Chao.

**Investigation:** Wen-Cheng Chao.

**Methodology:** Hsien-Fu Chiu, Yan-Chiao Mao.

**Writing – original draft:** Wei-Fan Ou, Wen-Cheng Chao.

**Writing – review & editing:** Wen-Cheng Chao.

## Abbreviations

Abbreviations: AIN = acute interstitial nephritis, AKI = acute kidney injury, ALT = alanine aminotransferase, ARDS = acute respiratory distress syndrome, AST = aspartate aminotransferase, ATN = acute tubular necrosis, CK = creatine kinase, Cr = creatinine, CRRT = continuous renal replacement therapy, CVVH = continuous veno-venous hemofiltration, CXR = chest X-ray, ECG = electrocardiogram, Hb = hemoglobin, HD = hemodialysis, IHD = intermittent hemodialysis, LDH = lactate dehydrogenase, PEEP = positive end-expiratory pressure, RNA-Seq = RNA-sequencing, SLED = sustained low-efficiency hemodialysis, TCVGH = Taichung Veterans General Hospital.
